# The Expression Patterns of Non-Coding RNAs in Periodontal Disease

**DOI:** 10.3390/dj12060159

**Published:** 2024-05-24

**Authors:** Dimitra Adamouli, Chrysa Marasli, Yiorgos A. Bobetsis

**Affiliations:** 1 Private Practice, 11527 Athens, Greece; dadamouli@uth.gr (D.A.); maraslic@med.uoa.gr (C.M.); 2 Department of Periodontology, School of Dentistry, National and Kapodistrian University of Athens, 11527 Athens, Greece

**Keywords:** noncoding RNAs, epigenetics, periodontal disease

## Abstract

During the last few decades there has been a growing interest in understanding the involvement of epigenetics in the pathogenesis and treatment of periodontal disease. Noncoding RNAs (ncRNAs), including microRNAs (miRNAs), long noncoding RNAs (lncRNAs), and circular RNAs (circRNAs), may serve as epigenetic modifiers affecting the expression of genes involved in the pathogenesis of inflammatory and autoimmune diseases. There is increasing evidence supporting the idea that the function of all three types of ncRNAs seems to be interdependent. LncRNAs can act as miRNA decoys, while circRNAs can act as miRNA sponges, leading to the re-expression of miRNA target genes. The purpose of this review is to evaluate the expression patterns of ncRNAs in periodontal disease. Studies demonstrate a positive correlation between miRNA expression and periodontitis; however, this cannot be claimed for lncRNAs and circRNAs, which appear to be differentially expressed in periodontitis patients. Several studies have also suggested utilizing ncRNAs as diagnostic and prognostic biomarkers in periodontitis, or even as potential therapeutic targets; Nevetheless, the evidence to support this is premature. Future well-designed research remains necessary to establish the functional role of ncRNAs in the evolution and progression of periodontal disease.

## 1. Introduction

Periodontitis is a chronic inflammatory disease characterized by the progressive destruction of the supportive tissues of the teeth [1]. A bacterial infection is considered the main cause of the disease; however, several environmental and genetic parameters may affect both the disease onset and progression [2]. Specifically, periodontal disease is initiated by the activation of the host’s inflammatory response against the bacterial stimuli. This response is mediated through the expression of a complex network of proinflammatory cytokines, which in turn lead to cellular activation. Obviously, various extrinsic and intrinsic factors that could modulate the host’s inflammatory response mechanisms would affect disease progression. Probably the most studied and recognized examples of such factors include smoking [3], diabetes mellitus [4], and gene polymorphisms [5].

In recent years, there has also been growing interest in understanding the involvement of epigenetics in the pathogenesis and treatment of periodontal diseases [6]. Epigenetics is the study of chemical modifications in DNA and its associated proteins that alter gene expression [7]. The epigenome is dynamically formed by genomic and environmental interactions asserting the unique individual phenotype [8,9]. The main epigenetic modification mechanisms include DNA methylation, histone modification, and gene regulation by nonprotein coding RNAs (ncRNAs).

NcRNAs are a class of RNAs without proteincoding functions that regulate gene expression and cell differentiation at the genomic and chromosomal levels [10,11,12,13]. According to their length, they are mainly divided into short-chain ncRNAs or microRNAs (miRNAs) and long noncoding RNAs (lncRNAs) [14,15]. MiRNAs are 17 to 25 nucleotides long and evolutionarily conserved, stemming from larger transcripts, while lncRNAs are linear RNAs with a length of more than 200 nucleotides [8,15,16].Circular RNAs (circRNAs)are considered a special class of ncRNAs that are different from linear RNAs.They are covalently closed lncRNAs, mainly encountered in the cytoplasm of eukaryotic cells [17].

Early studies comparing the expression profiles of ncRNAs from gingival tissues of healthy and periodontitis patients have revealed significant differences [18,19]. Specifically, using microarray technology, it was demonstrated that 159 miRNAs and 8925 lncRNAs were differentially expressed, indicating a possible role of these ncRNAs in the pathogenesis and development of periodontitis. Since then, many other studies have focused on the expression of specific ncRNAs in periodontal disease, in an attempt to identify potential targets for the treatment of periodontal disease or potential biomarkers for the diagnosis and assessment of periodontitis. In the present review, we summarize the current knowledge regarding the differences in the expression of ncRNAs among periodontitis and healthy individuals.

### 1.1. MiRNAExpression in Periodontal Disease

The studies that evaluated the expression of miRNAs in periodontitis patients are presented in Table 1. Most of the results derive from studies where the participants suffered from chronic periodontitis, while only a few included patients with aggressive periodontitis [20,21,22,23]. Most studies showed a positive correlation in the expression of specific miRNAs and the presence as well as progression of periodontal disease, while only a relatively small number linked miRNA downregulation with periodontitis [24,25,26,27,28,29,30,31].

The only two studies that compared the miRNA expression between chronic and aggressive periodontitis patients revealed no significant differences, although both groups demonstrated an increase in miRNA expression when compared to the periodontally healthy group [21,23].

Interestingly, the study by Fujimori et al., 2019 is the only study that aimed to investigate salivary miRNAs reflecting periodontal condition in chronic periodontitis. This cross-sectional pilot study included 120 participants with mild, moderate, or severe chronic periodontitis. Among the 84 miRNAstested, only hsa-miR-381-3p was significantly upregulatedin the severe periodontitis group. Moreover, as anticipated, there was also a significant correlation between the relative expression of this miRNA and the mean pocket depth [32]. In addition, the same group (Fujimori et al., 2021) also evaluatedmiRNA expression in relation to the progression of periodontitis. Specifically, in this two-year cohort study they assessed the expression of several miRNAs from the saliva in 120 patients undergoing supportive periodontal therapy [33]. According to this study, there was a positive correlation between the expression of several miRNAs and the progression of chronic periodontitis.

Some authors also evaluated how systemic diseases, such as diabetes mellitus [27,34,35,36], obesity [37,38,39], rheumatoid arthritis [40], and coronary heart disease [26,41], influence relative miRNA expression levels in patients with a healthy or diseased periodontium. Results from these studies have shown that miRNA expression was significantly upregulated in patients suffering from both periodontal disease and diabetes, rather than exclusively diabetes or periodontitis. Similar results are also observed for obese and coronary heart disease patients with periodontitis [26,37,38,39,41]. This could lead to the conclusion that the presence of certain systemic diseases synergistically affects the regulation of microRNAs related to periodontal disease. On the other hand, data were inconclusive in patients with chronic periodontitis with or without rheumatoid arthritis [40].

Smoking, a well-established risk factor for periodontal disease, has also been evaluated for its effect on miRNA expression in periodontitis patients. In the study of Öngöz Dede et al., 2022 the expression of six miRNAs in the saliva of smokers and nonsmokers with periodontal disease, before and after nonsurgical periodontal therapy, was determined [42]. Salivary miR-146a, miR-146b, miR142-3p, miR-155, and miR-203 gene expression was significantly upregulated in periodontitis patients compared to the control group both in smokers and nonsmokers. Moreover, a significant increase in miR-142-3p expression was detected in all groups of smokers compared to nonsmokers. Hence, the authors suggested that smoking may contribute to an increase in the levels of salivary miR-142-3p in periodontal health and disease. Finally, although there was a decrease in miRNA gene expression after periodontal treatment, this was not statistically significant.

Among the plethora of miRNAs examined, MiR-146a is the mostfrequently analyzed [22,26,36,41,43,44,45,46]. MiR-146a is involved in the expression of tumor necrosis factor-α (TNF-α), interleukin (IL)-b, and IL-6, and therefore is associated with the pathogenesis of several inflammatory diseases [42,46]. Its upregulation seems to play a functional role in periodontal disease by exhibiting a negative regulation of immune response [47]. Patients with chronic periodontitis exhibit up to a 32,6 timeshigher expression of this miRNA [48]. These findings are also in agreement with the results of the the larger studyin terms of sample size included in this review [45]. Only the study by Rovas et al. found that miR-146a-5p expression levels were significantly lower among patients with periodontal disease [40]. Other frequently studied miRNAs are miR-142-3p, miR-223, miR-155, miR-205, and miR-21. MiR-223 is a modulator in osteoclastogenesis, and its high expression in periodontal disease should be further examined as it might affect alveolar bone loss [49].

**Table 1:** Summary of publications evaluating miRNA expression in periodontal disease.

Reference	Type of Study	Cases	Type ofSample	Type of Periodontal Disease	Results
Uttamani et al., 2023 [24]	Cohort	CP, 27 Healthy, 27	Gingival biopsy	Chronic periodontitis 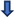	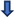 miR-26a-5p, FC: 0.38± 0.14;miR-26b-5p, FC:0.41 ± 0.21 in CP
Costantini et al., 2023 [50]	Cross-sectional	CP, 21 Healthy, 15	Gingival crevicular fluid	Chronic periodontitis	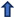 miRNA-103a-3p significant differences between mild and severe stages with a variation of 1.27-fold miRNA-23a-3pa fold change of 11.32 in patients with mild periodontitis, while in moderate and severe periodontal disease the expression levels were 1.9- and 1.4-fold, respectively miRNA-15a-5p, miRNA-223-3p, and miR-423-5p severe periodontitis 3.68-fold, in relation to patients with periodontitis in mild (0.39-fold) and moderate stages (0.45-fold)
Liu et al., 2022 [34]	Cross-sectional	G1: CP, 26 G2: T2DM, 24 G3: CP + T2DM,22 G4: healthy, 25	Serum and gingival crevicular fluid	Chronic periodontitis	miR-223, miR-200b high in G1 and G2, and higher in G3
Öngöz Dede et al.,2022 [42]	Cohort	G1: CP + nonsmokers, 15 G2: CP + smokers, 15 G3: gingivitis + nonsmokers, 15 G4: gingivitis + Smokers, 15 G5: periodontically healthy + nonsmokers, 15 G6: periodontically healthy + smokers, 15	Saliva	Chronic periodontitis/gingivitis	Salivary miR-146a, miR-146b, miR142-3p, miR-155, and miR-203in CP Smoking may contribute to an increase in the levels of salivary miR-142-3p in periodontal health and disease
Chaparro et al., 2022 [47]	Case–control	CP, no data Healthy, no data	Gingival biopsy	Chronic periodontitis	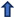 miRNA-20a, miRNA-30e, and miRNA-93 in CP
Mogharehabedet et al., 2022 [51]	Sectional	CP, 10 Healthy, 10	Blood sample	Chronic periodontitis	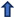 miR-155, FC:64.4 in CP
Huang and Jia, 2022 [25]	Cohort	CP, 76 Healthy, 71	Gingival crevicular fluid PDLCs	Chronic periodontitis	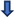 miR-28-5p in CP
Zhu and Zhong, 2022 [52]	Cohort	CP, 80 Healthy, 100	Gingival crevicular fluid	Chronic periodontitis	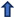 miR-30b-3p and miR-125b-1-3p may be associated with the pathogenesis of periodontitis
Rovas et al., 2022 [40]	Cross-sectional	CP, 134 Healthy periodontically with or without RA, 76	Gingival crevicular fluid	Chronic periodontitis	miR-140-3p, miR-145-5p, and miR-146a-5p in CP in pt with or without RA
	t				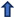
Rovas et al., 2021 [53]	Cross-sectional	CP, 30 Healthy, 31 Diagnosed with RA, 25	Gingival tissue GCF Saliva Plasma	Chronic periodontitis	miR-199a-5p, miR-483-5p, miR3198, and miR-4299 associated with the presence and/or severity of PD
Mahendra et al., 2021 [26]	Cohort	GP+CAD, 25 CP, 25 Healthy, 25	Oral subgingival plaque	Generalized periodontitis	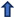 miRNA-146a and 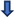 miRNA-126 in the GP + CAD group
Fujimori et al., 2021 [33]	Cohort	Patients who underwent supportive periodontal therapy, 120	Saliva	Chronic periodontitis	hsa-miR-5571-5p, FC:3.70; hsa-let-7f-5p, FC:7.90; hsa-miR-99a-5p, FC:2.71; hsa-miR-28-5p, FC:6.86; and hsa-miR-320d, FC:4.51 were associated with periodontitis progression
Elazazy et al., 2021 [27]	Cross-sectional	G1:CP, 20 G2: type 2 diabetes CP, 20 G3: healthy, 20	Serum and gingival crevicular fluid	Chronic periodontitis	miR-223 and miR-200bin G1 and G2 miR-203in G1 and G2
Du et al., 2021 [28]	Cohort	CP, 72 Healthy, 50	Gingival crevicular fluid	Chronic Periodontitis	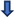 miR-1226 in CP
Lee et al., 2020 [20]	Pilot case–control	AP, 4 Healthy, 4	Saliva	Aggressive periodontitis	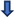 hsa-let-7a-5p, FC:3.81; hsa-let-7f-5p, FC:3.40; hsa-miR-181b-5p, FC:5.91; and hsa-miR-23b-3p, FC:3.66 in AP
Gonçalves Fernandes et al., 2020 [29]	Cohort	LAP, no data Healthy, no data	Blood sample	Localized aggressive periodontitis	miRNAs miR-9-5p, FC:2.23; miR-155-5p, FC:2.01; 203a-3p, FC:2.17; miR-147a, FC:2.11; miR-182-5p, FC:2.46; and miR-183-5p, FC:2.48 in LAP
Jia et al., 2020 [54]	Cross-sectional	CP, 24 Healthy, 18	Gingival biopsy	Chronic periodontitis	miRNA 210 in CP
Al-Rawi et al., 2020 [43]	Pilotcross-sectional	DM, 24 Healthy, 29 Each group was subdivided into periodontally healthy or having periodontitis, specific data unknown	Saliva	Chronic periodontitis	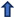 miRNAs-146a/b, FC:4.3/12.3; miR-155, FC:13.7 for periodontitis diabetic pt, and miR-203, FC:5.9 higher in patients with CP and/or DM
Nisha et al., 2019 [55]	Cross-sectional	CP, 16 Healthy, 16	Saliva	Chronic periodontitis	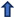 miR-143-3pin CP, FC:5.82
Ou et al., 2019 [35]	Cross-sectional	CP, 45 Healthy, 50 DM, 40 CP+ DM, 63	Gingival biopsy +GCF	Chronic periodontitis	miR-214in diabetes- associated periodontitis patients
Amaral et al., 2019 [21]	Cross-sectional	CP, 9 AP, 9 Healthy, 66	Gingival biopsy	Chronic periodontitis and aggressive periodontitis	No differences in the miRNA expression profiles between CP and AP 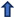 hsa-miR-1274b, hsa-let-7b-5p, hsa-miR-24-3p, hsa-miR-19b-3p, hsa-miR-720, hsa-miR-126-3p, hsa-miR-17-3p, and hsa-miR-21-3p
Zhang et al., 2019 [56]	Cross-sectional	CP, 29 Healthy, 21	Gingival crevicular fluid	Chronic periodontitis	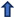 miR-23a in CP potential biomarker
Zhao et al., 2019 [44]	Cross-sectional	CP, 38 Healthy, 30	Serum	Chronic periodontitis	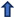 miRNA-146ain CP
Fujimori et al., 2019 [32]	Cross-sectional	G1: no/mild CP, 26 G2: moderate CP, 58 G3: severe CP, 36	Saliva	Chronic periodontitis	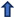 miRNA hsa-miR-381-3p in CP, FC:3,63
Bagavad Gita et al., 2019 [41]	Case–control	G1: CHD no CP, 66 G2: CHD + CP, 66 G3: healthy + CP, 66 G4: healthy no CP, 66	Blood sample	Chronic periodontitis	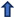 miRNA 146 overexpressed in G1, G2, and G3 with no significant differences between them
Naqvi et al., 2019 [37]	Cross-sectional	G1: Obesityno/+ CP, 14 G2: Healthy no/+ CP, 14	Gingival biopsy	Chronic periodontitis	Significant differences in miRNA expression levels between healthy and obese subjects and periodontal and nonperiodontal patients
Yoneda et al., 2019 [57]	Case-control	CP, 30 Healthy, 30	Blood sample	Chronic periodontitis	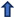 miR-555, FC:1.85; miR-130a-5p, FC:1.71; miR-664a-3p, FC:1.54; miR-501-5p, FC:1.57; miR-6770-5p, FC:0.65; miR-4717-5p, FC:0.64; miR21-3p, FC:0.63 in CP
Micó-Martínez et al., 2018 [30]	Cross-sectional	CP, 9 Healthy, 9	Gingival crevicular fluid	Chronic periodontitis	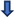 miRNA 1226, FC:15.8 in CP
Venugopal et al., 2018 [58]	Cross-sectional	CP, 100 Healthy, 100	Gingival biopsy	Chronic periodontitis	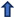 Let/a, miRNA-21, FC:2; miRNA-100 in CP
Li et al., 2019 [59]	Cross-sectional	CP, 16 Healthy, 16	Gingival biopsy	Chronic periodontitis	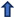 miRNA 144 in CP
Ghotloo et al., 2019 [22]	Cross-sectional	AP, 18 Healthy, 10	Gingival biopsy	Aggressive periodontitis	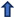 miRNA 146, FC:17.8 in AP
Radović et al., 2018 [36]	Cohort	G1: T2DMno CP, 24 G2: T2DM+ CP, 24 G3: healthy no CP, 24 G4: healthy + CP, 24	Gingival crevicular fluid	Chronic periodontitis	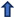 miRNA 146 and 155 in CP
Saito et al., 2017 [23]	Cross-sectional	CP, 7 AP, 2 Healthy, 11	Gingival crevicular fluid	Chronic and aggressive periodontitis	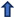 miR-223-3p, miR-203a, and miR-205-5p in CP and AP
Na et al., 2016 [31]	Cross-sectional	CP, no data Healthy, no data	Gingival biopsy	Chronic periodontitis	miR-128, miR-34a, and miR-381in CP miR-15b, miR211, miR-372, and miR-656 in CP
Motedayyen et al., 2015 [48]	Cross-sectional	CP, 20 Healthy, 10	Gingival biopsy	Chronic periodontitis	miRNA 146a expression 32,6 times greaterin CP
Kalea et al., 2015 [38]	Cross-sectional	Normal-weight subjects with severe periodontitis, 17 Obese subjects with severe periodontitis, 19	Gingival biopsy	Severe periodontitis CP or AP	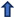 miR200b, FC:1.27 in obese periodontitis subjects
Ogata et al., 2014 [60]	Cross-sectional	CP 3 Healthy 3	Gingival biopsy	Chronic periodontitis	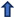 miRNA 150, 223, and 200b, FC: >2.72 in CP
Kadkhodazadeh et al., 2013 [45]	Cohort study	CP 75 PI:38 Healthy 84	Blood sample	Chronic periodontitis Peri-implantitis	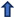 miRNA 146a and 499 in CP and PI
Perri et al., 2012 [39]	Cross-sectional	G1: periodontically healthy + non-obese 5 G2: CP + non-obese, 5 G3: periodontically healthy + obese, 5 G4: CP + obese, 5	Gingival biopsy	Chronic periodontitis	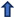 G3: miR-18a, FC:1.5; miR-30e, FC:2.1 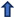 G2: miR-30e, FC:4.4; miR-106b, FC:4.9 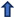 G4: miR-15a, FC:2.7; miR-18a, FC:1.5; miR-22, FC:3.1; miR-30d, FC:2.2; miR-30e, FC:4.9; miR-103, FC:1.5; miR-106b, FC:6.4; miR-130a, FC:4.6; miR-142-3p, FC:5.3; miR-185, FC:3.6; and miR-210, FC:2.3
Lee et al., 2011 [61]	Cross-sectional	No data	Gingival biopsy	Chronic periodontitis	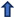 miR-181b, miR-19b, miR-23a, miR-30a, miR-let7a, and miR-301a
Stoecklin-Wasmer et al., 2012 [18]	Cross-sectional	CP, 86	Gingival biopsy	Chronic periodontitis	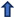 hsa-miR-451, FC:2.63; hsa-miR-223, FC:2.53; hsa-miR-486-5p, FC:2.46; and hsa-miR-3917, FC:2.08 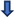 hsa-miR-1246, FC:0.33; hsa-miR-1260, FC:0.44; hsa-miR-141, FC:0.46; hsa-miR-1260b, FC:0.44; hsa-miR-203 and hsa-miR-210, FC:0.46; and hsa-miR-205*, FC:0.49
Xie et al., 2011 [46]	Cross-sectional	CP, 10 Healthy, 10	Gingival biopsy	Chronic periodontitis	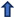 hsa-miRNA-146a, hsa-miRNA-146b, and hsa-miRNA-155

Abbreviations: CP: chronic periodontitis; GP: generalized periodontitis, AP: aggressive periodontitis; LAP: localized aggressive periodontitis; T2DM: type 2 diabetes mellitus; DM: diabetes mellitus; GCF: gingival crevicular fluid; CHD: coronary heart disease; CAD: coronary artery disease; PI: peri-implantitis; PDLCs: periodontal ligament cells; RA: rheumatoid arthritis; G: group; and FC: fold change, 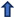 upregulation, 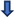 downregulation.

### 1.2. LncRNAExpression in Periodontal Disease

The studies that evaluated the expression of lncRNAs in periodontitis patients are presented in Table 2. As with miRNAs, most of the studies are performed in patients suffering from chronic periodontitis;however, in most cases the severity of periodontal disease is not specified. Thus, from this set of studies it is not safe to link disease severity and progression with the level of lncRNA expression. Moreover, there is great variability in the lncRNAs evaluated, which renders comparisons among studies challenging. In addition, the interpretation of the outcomes is complex considering the inconsistent results. Thus, 9 of the studies show that the downregulation of the expression of specific lncRNAs is linked to periodontal disease [62,63,64,65,66,67,68,69,70], while 7 studies associate periodontitis with the upregulation of the expression of lncRNAs [71,72,73,74,75,76,77]. The best example includes MALAT1. Although this is the most frequently analyzed lncRNA, it is evaluated only in three studies [65,74,77]. From these studies, Li et al., 2020 [74] found an increase in the expression of MALAT1 in gingival biopsies derived from patients with periodontitis stage III grade C. Similarly, Chen et al., 2019 [77] also concluded that MALAT1 was upregulated in periodontal ligament cells from chronic periodontitis patients. On the other hand, the study by Gholami et al., 2020 [65] did not support these findings and showed no difference in MALAT1 expression levels from gingival biopsies and blood samples among healthy individuals and chronic periodontitis patients.

Since lncRNAs may interact with miRNAs, several investigators have also tried to assess the relationship between the expression profiles of certain miRNAs and lncRNAs [67,69]. Interestingly, those two studies concluded that lncRNA expression is inversely correlated with miRNA expression. Specifically, the expression of miR-27a-3p [67] and miR-142-3p [69] was increased in periodontitis patients, while the respective expression of the lncRNAs (MEG3, and FDG5-AS1) was downregulated.

Finally, another interesting finding was that one study [73] showed that the expression of lncRNA PACER was significantly higher in blood samples of female subjects when compared to male subjects with chronic periodontitis, concluding that there may be sex-specific functions to lncRNA levels and inflammatory control.

**Table 2:** Summary of publications evaluating lncRNA expression in periodontal disease.

Reference	Type of Study	Cases	Type of Sample	Type of Periodontal Disease	Results
Zhang et al., 2022 [71]	Case–control	CP, 28 Healthy, 20	Gingival biopsy	Chronic periodontitis	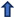 LncRNA NEAT1 in CP
Yang et al., 2022 [62]	Case–control	P, 7 Healthy, 8	Gingival biopsy	Not specified	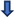 LncRNA GAS5 in P downregulated by 50%
Han et al., 2022 [63]	Case–control	P, 12 Healthy, 10	Gingival biopsy	Not specified	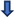 LncRNA SNHG5 in P
					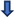
Wang et al., 2021 [64]	Case–control	SP, 30 Healthy, 30	PDLCs from extracted teeth	Stable periodontitis	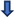 LncRNA DCST1-AS1 in SP
Zhou et al., 2021 [72]	Case–control	P, 47 Healthy, 19	Gingival biopsy	Not specified	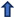 LncRNA LINC01126 in P1080 lncRNAs significantly differentially expressed with fold changes greater than 1.5
Sayad et al., 2020 [73]	Case–control	Gingival biopsy: CP, 30 Healthy, 30 Blood samples: CP, 23 Healthy, 18	Gingival biopsy and blood sample	Chronic periodontitis (stages II to IV)	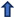 LncRNA PACER in blood samples in CP especially in female subjects
Li et al., 2020 [74]	Case–control	CP, 20 Healthy, 20	Gingival biopsy	Chronic periodontitis (stage III, grade C)	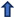 LncRNA MALAT1 in CP
Guo et al., 2020 [75]	Case–control	P, 5 Healthy, 5	PDLCs from extracted teeth	Not specified	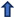 LncRNA H19 in P1080 lncRNAs significantly differentially expressed with fold changes greater than 1.5
Gholami et al., 2020 [65]	Case–control	CP, 30 Healthy, 30	Gingival biopsy and blood sample	Chronic periodontitis	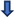 LncRNA ANRIL in blood samples in CP Expression of lncRNA MALAT1 is the same in both groups
Dong et al., 2020 [66]	Case–control	P, 25 Healthy, 25	PDLCs from extracted teeth	Not specified	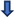 LncRNA MEG3 in P
Wang et al., 2019 [76]	Cohort	P, 80 Healthy, 66	Blood sample	Not specified	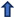 LncRNA AWPPH in P Correlated with high recurrence rate
Liu et al., 2019 [67]	Case–control	P, 20 Healthy, 20	PDLCs from extracted teeth	Not specified	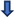 LncRNA MEG3 in P, while miR-27a-3p was upregulated
Liu et al., 2019 [68]	Case–control	P, 34 Healthy, 34	PDLCs from extracted teeth	Not specified	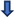 LncRNA PTCSC3 in P
Chen et al., 2019 [77]	Case–control	CP, 12 Healthy, 12	PDLCs from extracted teeth	Chronic periodontitis	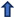 LncRNA MALAT1 in CP
Chen et al., 2019 [69]	Case–control	CP, 26 Healthy, 20	Gingival biopsy	Chronic periodontitis	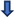 LncRNA FGD5-AS1 in CP, while miR-142-3p was upregulated
Wang et al., 2016 [70]	Case–control	SP, 10 Healthy, 10	PDLCs from extracted teeth	Stable periodontitis	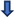 LncRNA POIR in SP
Zou et al., 2015 [19]	Cross-sectional	CP, 30 Healthy, 15	Gingival tissues	Chronic periodontitis	8925 differently expressed lncRNAs 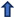 4313 lncRNAs 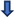 4612 lncRNAs

Abbreviations: CP: chronic periodontitis; P: periodontitis; SP: stable periodontitis; PDLCs:periodontal ligament cells; NEAT1:nuclear paraspeckle assembly transcript 1; GAS5:growth arrest specific transcript 5; SNHG5:small nucleolar RNA host gene 5; PACER: p50-associated COX-2 extragenic RNA; MALAT1:metastasis-associated lung adenocarcinoma transcript 1; ANRIL: antisense noncoding RNA in the INK4 locus; MEG3:maternally expressed gene 3;and PTCSC3:papillary thyroid carcinoma susceptibility candidate 3, 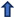 upregulation, 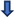 downregulation.

### 1.3. CircRNAExpression in Periodontal Disease

The studies that evaluated the expression of circRNAs in periodontitis patients are presented in Table 3. Most of these studies were designed to explore, in vitro, the role of circRNAs in periodontitis and secondarily evaluated the levels of expression of circRNAs in periodontitis patients. The data suggest no clear pattern in the expression of various circRNAs. All three studies that evaluated the expression of Circ_0099630 found upregulation in patients with periodontitis [78,79,80]. In the study by Wang et al. [78], circ_0099630 upregulation was correlated with the downregulation of miR_212-5p, while in the study by Zhao et al. [80], miR-940 was downregulated.

On the contrary, in two other studies the expression of various circRNAs was downregulated [81,82] in periodontitis patients, with the authors suggesting a possible protective action of these circRNAs against periodontitis. Finally, DNA sequencing in the study of Li et al. [83] showed that 1304 circRNAs were differentially expressed in chronic periodontitis patients.

All these findings should be interpreted with caution due to the small number of studies, the small sample size of the included studies, and the lack of a clear definition and characterization of periodontal disease severity.

**Table 3:** Summary of publications evaluating circRNA expression in periodontal disease.

Reference	Type of Study	Cases	Type of Sample	Type of Periodontal Disease	Results
Wang et al.2023 [78]	Case control	P, 10 Healthy, 10	PDLCs from extracted	Not specified	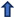 circ_0099630 in P 3-fold higher
Pan et al. 2022 [84]	Case–control	P, 30 Healthy, 30	PDLCs from extracted teeth	Not specified	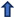 circ_0138959 in P
Wei et al. 2022 [79]	Case–control	P, 60 Healthy, 20	hPLFs from extracted teeth	Not specified	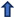 circ_0099630 in P
Zhao et al. 2022 [80]	Case–control	P, 26 Healthy, 21	Periodontal tissues	Not specified	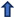 circ_0099630 in P linked with lower miR-940 expression
Wang et al. 2021 [81]	Case–control	P, 21 Healthy, 21	Gingival tissues	Not specified	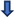 circ_0081572 in P
Yu et al. 2021 [85]	Case–control	P, 20 Healthy, 10	PDLCs from extracted teeth	Not specified	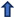 circ_002284 (circMAP3K11) in P
Zheng et al. 2020 [86]	Case–control	CP, 6 Healthy, 6	PDLCs from extracted teeth	Chronic periodontitis	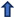 circ_0003489 (circCDK8) in CP 2-fold higher
Li et al. 2019 [83]	Case–control	CP, 4 Healthy, 4	Gingival tissues	Chronic periodontitis	1304circRNAs were differentially expressed in CPby greater than a 2-fold change 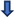 circ_0062491 and 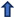 circ_0095812in CP
Wang et al., 2019 [82]	Case–control	CP, 10 Healthy, 11	PDLCs from extracted teeth	Chronic periodontitis	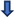 circRNA CDR1as in CP

Abbreviations: CP: chronic periodontitis; P: periodontitis; PDLCs:periodontal ligament cells, 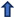 upregulation, 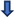 downregulation.

### 1.4. Interactions of ncRNAs in Periodontitis

Since their discovery, miRNAs have attracted more attention than their longer noncoding counterparts; therefore, the number of studies concerning miRNAs is significantly higher than those for lncRNAs and circRNAs. Overall, there seems to be a positive correlation between miRNA expression and periodontal disease. MiRNAs play a crucial role in the pathogenesis of periodontitis by regulating various signaling pathways involved in inflammatory responses, bone remodeling, and stem cell differentiation [33,87,88,89,90].

Hence, several miRNAs, such as miR-146a, miR-155, and miR-223, regulate various aspects of the innate and adaptive immune responses against periodontal pathogens, including the targeting components of signaling pathways like NF-κB [87,88]. MiR-99a-5p may affect periodontitis progression through the MAPK signaling pathway, which is involved in regulating cellular processes like inflammation, proliferation, and differentiation [33]. Additionally, miR-132 and miR-222 can regulate the osteogenic differentiation of periodontal ligament stem cells (PDLSCs) by modulating the Wnt/β-catenin and BMP signaling pathways, respectively [89]. MiR-146a, miR-155, and miR-223 can also modulate the signaling pathways involved in osteoclastogenesis and osteoblast differentiation, which are key processes in periodontal bone loss [90].

However, there is increasing evidence supporting that the function of miRNAs is regulated, at least in part, by the other two ncRNAs, ielncRNAs and cirRNAs. Probably the most studied interaction between ncRNAs in relation to periodontitis includes the ability of some lncRNAs and circRNAs to act as sponges/decoys for miRNAs through miRNA reaction elements. Hence, they both serve as competitive endogenous RNAs (ceRNAs) against miRNAs for binding sites in mRNA [91,92,93]; therefore, lncRNAs can modulate signaling pathways central to periodontitis pathogenesis, such as Wnt, NF-κB, and MAPK, by acting as ceRNAs that sponge miRNAs or by directly targeting pathway components. For example, the lncRNA POIR acts as a sponge for miR-182, which binds to the 3’ UTR of FOXO1. FOXO1 inhibits the canonical Wnt signaling pathway, suggesting that POIR lncRNA may regulate Wnt signaling in periodontitis [94,95]. Moreover, the overexpression of the lncRNA OIP5-AS1 is thought to inhibit the lipopolysaccharide (LPS)-induced inflammatory response in human periodontal ligament stem cells (PDLSCs) by sponging miR-92a-3p, which likely involves the NF-κB pathway [96]. In addition, differentially expressed lncRNAs in periodontitis are predicted to target genes enriched in the MAPK signaling pathway, suggesting their potential role in regulating this pathway [33]. Finally, the relationship among miRNAs, lncRNAs, and circRNAs in periodontal disease involves a network of interactions that regulate the osteogenic differentiation of PDLCs/PDLSCs [97,98], which is important to alveolar bone regeneration. It becomes clear that the interplay between these noncoding RNAs represents an emerging area of research in understanding the molecular mechanisms underlying periodontitis. Therefore, looking only into the expression levels of only one ncRNA may be misleading.

### 1.5. Significance of Sample Source in ncRNA Expression

The ncRNA profiling occurred from the analysis of samples originating from various sources, such as blood, serum, plaque, gingival biopsies, gingival crevicular fluid (GCF), saliva, and periodontal ligament cells. This may raise a question as to whether the results are comparable, since the proximity to the diseased periodontal tissues and the effect of systemic influences may differ. It is reasonable to suggest that gingival biopsies present tissue sensitivity and specificity, since they include the diseased site. GCF is the sample source with closer proximity to the gingival tissues and becomes an inflammatory exudate containing a great number of proteins and peptides deriving from host inflammatory cells, oral bacteria, and structural cells of the periodontium and periodontal pocket [99]. Therefore, the analysis of the GCF constituents is considered to reflect the disease status of periodontitis [100]. On the other hand, saliva, serum, and blood samples derive from more distant sites and thus could underestimate the actual level of disease in the gingiva. Therefore, fold changes in the expression of ncRNAs observed from different sample sources may not be directly comparable. In a recent systematic review and meta-analysis by Asa’ad et al., 2020 [101] the authors attempted to assess this issue; however, a statistical analysis was not possible for evaluating differences between methods of sample collection as tissue biopsies were the procedure of choice in the majority of the included studies.

In addition, the direct comparison of the expression levels of ncRNAs among different sample sources in the same population was very scarce. In the study by Elazazy et al., 2021 [27] there was a significant overexpression of miR-223 in both the serum and GCF of chronic periodontitis patients with and without diabetes type 2 as compared to the controls. Furthermore, in both patient groups the GCF levels of miR-223 were significantly elevated in comparison with the corresponding serum levels. On the other hand, miR-200b was also overexpressed in both the serum and GCF of both patient groups, although expression was significantly higher in the serum. Since elevated levels of miR-200b are involved in the increased pancreatic β-cell apoptotic rate [102], the authors suggested that miR-200b is more implicated in the pathomechanism of diabetes;therefore, it is possible that systemic diseases may affect the expression of specific ncRNAs, which may become more obvious in serum samples.

Finally, in the study by Rovas et al., 2021 [53] the authors evaluated the expression of miRNAs in gingival tissue, GCF, saliva, and plasma from patients with or without periodontitis. The relative quantities of selected miRNAs (miR-199a-5p, miR-483- 5p, miR-3198, and miR-4299) were significantly different between GCF, saliva, and plasma samples. The level of miR-3198 was 12.5-fold higher in GCF than in saliva and 85.8-fold higher in plasma (*p*< 0.001). Meanwhile, the miR-199a-5p level was 139.7-fold higher in plasma compared to saliva and 6.6-fold higher than in GCF (*p*< 0.001). Again, this study highlights the differences in the expression of miRNAs depending upon the sample source and type of miRNA.

### 1.6. NcRNAExpression and Its Use as a Biomarker for Periodontitis

Since miRNAs display stability in biofluids, and can be easily detected in tissue samples, investigators have considered them potential biomarkers for the diagnosis and assessment of periodontitis;therefore, a number of studies support the idea that miR-223, miR-200b, miR-28-5p, miR-23a, miR-146a, hsa-miR-381-3p, miR-1226, miRNA-155, miR143-3p, hsa-miR-664a-3p, hsa-miR-501-5p, and hsa-miR-21-3p could serve as biomarkers for the diagnosis and assessment of periodontitis [97,98], while miRNA-146a and miRNA-126 may serve as risk biomarkers for coronary artery disease and generalized periodontitis [26].

In addition, a small number of clinical studies have also explored the diagnostic and prognostic value of lncRNAs in chronic periodontitis. A notable example is the study by Wang et al. [76] in which the levels of AWPPH lncRNA before and after periodontal treatment were measured, and investigators found that high levels of AWPPH had high prognostic value for chronic periodontitis recurrence.

### 1.7. Limitations of the Studies

All aforementioned studies compared the expression of ncRNAs between periodontitis patients and healthy individuals;however, not all of them used the same classification system to define periodontitis. The majority of studies categorized patients according to the classification proposed in the International Workshop held in 1999, but the most recent studies employed the latest classification of 2017 [103,104]. Moreover, specific clinical measurements and indices concerning periodontal status are missing in most of the studies;therefore, it is difficult to link the expression of certain ncRNAs to the severity of the disease. In addition, in almost all studies patients with periodontital disease were pooled into one diseased group irrespective of the severity of periodontitis. This may have a significant impact on the outcomes of the studies, since milder periodontitis may have a washout effect.

Another limitation, regarding mainly the lncRNAs and circRNAs, is the relatively small number of studies available. This in combination with the wide variability in ncRNAs examined, which in most studies do not overlap, weakening the association between specific ncRNAs and periodontitis and not permitting the extraction of solid conclusions. Finally, there seems to be significant variation in terms of the sample size among studies, ranging from 4 to 256 participants. This aspect should be taken into consideration when interpreting the results, especially those of the smaller studies.

## 2. Conclusions

In conclusion, the altered expression of ncRNAs was identified in periodontitis patients, highlighting their potential role in disease pathogenesis and treatment., In general, there seems to be a positive correlation between miRNA expression and periodontal disease, with miR-146a and miRNA-142-3p being consistently upregulated in patients with periodontitis; therefore, it is reasonable to suggest that these two miRNAs may have the best potential to serve as diagnostic biomarkers for periodontal disease activity. Regarding the lncRNAs and circRNAs, the available evidence does not support a clear expression pattern in periodontitis; however, due to methodological limitations, these outcomes should be interpreted with caution. Larger, well-designed studies using uniform definitions for periodontitis and examining similar ncRNAs seem necessary for the future.

## References

[1] Herrera D., Sanz M., Kebschull M., Jepsen S., Sculean A., Berglundh T., Papapanou P.N., Chapple I., Tonetti M.S., EFP Workshop Participants and Methodological Consultant (2022). Treatment of stage IV periodontitis: The EFP S3 level clinical practice guideline. J. Clin. Periodontol..

[2] Bartold P.M. (2018). Lifestyle and periodontitis: The emergence of personalized periodontics. Periodontol. 2000.

[3] Leite F.R.M., Nascimento G.G., Scheutz F., López R. (2018). Effect of Smoking on Periodontitis: A Systematic Review and Meta-regression. Am. J. Prev. Med..

[4] Preshaw P.M., Alba A.L., Herrera D., Jepsen S., Konstantinidis A., Makrilakis K., Taylor R. (2012). Periodontitis and diabetes: A two-way relationship. Diabetologia.

[5] Zhang J., Sun X., Xiao L., Xie C., Xuan D., Luo G. (2011). Gene polymorphisms and periodontitis. Periodontol. 2000.

[6] Barros S.P., Offenbacher S. (2014). Modifiable risk factors in periodontal disease: Epigenetic regulation of gene expression in the inflammatory response. Periodontol. 2000.

[7] Larsson L. (2017). Current Concepts of Epigenetics and Its Role in Periodontitis. Curr. Oral. Health Rep..

[8] Bayarsaihan D. (2011). Epigenetic mechanisms in inflammation. J. Dent. Res..

[9] Razzouk S., Termechi O. (2013). Host genome, epigenome, and oral microbiome interactions: Toward personalized periodontal therapy. J. Periodontol..

[10] Gareev I., Beylerli O., Aliev G., Pavlov V., Izmailov A., Zhang Y., Liang Y., Yang G. (2020). The Role of Long Non-Coding RNAs in Intracranial Aneurysms and Subarachnoid Hemorrhage. Life.

[11] Beylerli O., Khasanov D., Gareev I., Valitov E., Sokhatskii A., Wang C., Pavlov V., Khasanova G., Ahmad A. (2021). Differential non-coding RNAs expression profiles of invasive and non-invasive pituitary adenomas. Noncoding RNA Res..

[12] Gareev I., Beylerli O., Yang G., Izmailov A., Shi H., Sun J., Zhao B., Liu B., Zhao S. (2021). Diagnostic and prognostic potential of circulating miRNAs for intracranial aneurysms. Neurosurg. Rev..

[13] Sun J., Sun Z., Gareev I., Yan T., Chen X., Ahmad A., Zhang D., Zhao B., Beylerli O., Yang G. (2021). Exosomal miR-2276-5p in Plasma Is a Potential Diagnostic and Prognostic Biomarker in Glioma. Front. Cell Dev. Biol..

[14] Wei J.W., Huang K., Yang C., Kang C.S. (2017). Non-coding RNAs as regulators in epigenetics (Review). Oncol. Rep..

[15] Marques-Rocha J.L., Samblas M., Milagro F.I., Bressan J., Martínez J.A., Marti A. (2015). Noncoding RNAs, cytokines, and inflammation-related diseases. FASEB J..

[16] Gomez R.S., Dutra W.O., Moreira P.R. (2009). Epigenetics and periodontal disease: Future perspectives. Inflamm. Res..

[17] Beermann J., Piccoli M.T., Viereck J., Thum T. (2016). Non-coding RNAs in Development and Disease: Background, Mechanisms, and Therapeutic Approaches. Physiol. Rev..

[18] Stoecklin-Wasmer C., Guarnieri P., Celenti R., Demmer R.T., Kebschull M., Papapanou P.N. (2012). MicroRNAs and their target genes in gingival tissues. J. Dent. Res..

[19] Zou Y., Li C., Shu F., Tian Z., Xu W., Xu H., Tian H., Shi R., Mao X. (2015). lncRNA expression signatures in periodontitis revealed by microarray: The potential role of lncRNAs in periodontitis pathogenesis. J. Cell Biochem..

[20] Uttamani J.R., Naqvi A.R., Estepa A.M.V., Kulkarni V., Brambila M.F., Martínez G., Chapa G., Wu C.D., Li W., Rivas-Tumanyan S. (2023). Downregulation of miRNA-26 in chronic periodontitis interferes with innate immune responses and cell migration by targeting phospholipase C beta 1. J. Clin. Periodontol..

[21] Costantini E., Sinjari B., Di Giovanni P., Aielli L., Caputi S., Muraro R., Murmura G., Reale M. (2023). TNFα, IL-6, miR-103a-3p, miR-423-5p, miR-23a-3p, miR-15a-5p and miR-223-3p in the crevicular fluid of periodontopathic patients correlate with each other and at different stages of the disease. Sci. Rep..

[22] Liu L., Xiao Z., Ding W., Wen C., Ge C., Xu K., Cao S. (2022). Relationship between microRNA expression and inflammatory factors in patients with both type 2 diabetes mellitus and periodontal disease. Am. J. Transl. Res..

[23] Öngöz Dede F., Gökmenoğlu C., Türkmen E., Bozkurt Doğan Ş., Ayhan B.S., Yildirim K. (2023). Six miRNA expressions in the saliva of smokers and non-smokers with periodontal disease. J. Periodontal Res..

[24] Chaparro A., Lozano M., Gaedechens D., López C., Albers D., Hernández M., Pascual A., Nart J., Irarrazabal C.E. (2022). Exploring the Expression of Pro-Inflammatory and Hypoxia-Related MicroRNA-20a, MicroRNA-30e, and MicroRNA-93 in Periodontitis and Gingival Mesenchymal Stem Cells under Hypoxia. Int. J. Mol. Sci..

[25] Mogharehabed A., Yaghini J., Aminzadeh A., Rahaiee M. (2022). Comparative evaluation of microRNA-155 expression level and its correlation with tumor necrotizing factor α and interleukin 6 in patients with chronic periodontitis. Dent. Res. J..

[26] Huang P., Jia L. (2022). MicroRNA-28-5p as a potential diagnostic biomarker for chronic periodontitis and its role in cell proliferation and inflammatory response. J. Dent. Sci..

[27] Zhu J., Zhong Z. (2022). The expression and clinical significance of miR-30b-3p and miR-125b-1-3p in patients with periodontitis. BMC Oral. Health.

[28] Rovas A., Puriene A., Snipaitiene K., Punceviciene E., Buragaite-Staponkiene B., Matuleviciute R., Butrimiene I., Jarmalaite S. (2022). Gingival crevicular fluid microRNA associations with periodontitis. J. Oral. Sci..

[29] Rovas A., Puriene A., Snipaitiene K., Punceviciene E., Buragaite-Staponkiene B., Matuleviciute R., Butrimiene I., Jarmalaite S. (2021). Analysis of periodontitis-associated miRNAs in gingival tissue, gingival crevicular fluid, saliva and blood plasma. Arch. Oral. Biol..

[30] Mahendra J., Mahendra L., Fageeh H.N., Fageeh H.I., Ibraheem W., Abdulkarim H.H., Kanakamedala A., Prakash P., Srinivasan S., Balaji T.M. (2021). miRNA-146a and miRNA-126 as Potential Biomarkers in Patients with Coronary Artery Disease and Generalized Periodontitis. Materials.

[31] Fujimori K., Yoneda T., Tomofuji T., Ekuni D., Azuma T., Maruyama T., Sugiura Y., Morita M. (2021). Detection of Salivary miRNAs That Predict Chronic Periodontitis Progression: A Cohort Study. Int. J. Environ. Res. Public Health.

[32] Elazazy O., Amr K., Abd El Fattah A., Abouzaid M. (2021). Evaluation of serum and gingival crevicular fluid microRNA-223, microRNA-203 and microRNA-200b expression in chronic periodontitis patients with and without diabetes type 2. Arch. Oral. Biol..

[33] Du Y., Qi Y.S., Chen H., Shen G. (2021). The expression and clinical significance of miR-1226 in patients with periodontitis. BMC Oral. Health.

[34] Lee N.H., Lee E., Kim Y.S., Kim W.K., Lee Y.K., Kim S.H. (2020). Differential expression of microRNAs in the saliva of patients with aggressive periodontitis: A pilot study of potential biomarkers for aggressive periodontitis. J. Periodontal Implant. Sci..

[35] Gonçalves Fernandes J., Morford L.A., Harrison P.L., Kompotiati T., Huang H., Aukhil I., Wallet S.M., MacchionShaddox L. (2020). Dysregulation of genes and microRNAs in localized aggressive periodontitis. J. Clin. Periodontol..

[36] Jia S., Yang X., Yang X., Zhang F. (2020). MicroRNA-210 protects against periodontitis through targeting HIF-3α and inhibiting p38MAPK/NF-κB pathway. Artif. Cells Nanomed. Biotechnol..

[37] Al-Rawi N.H., Al-Marzooq F., Al-Nuaimi A.S., Hachim M.Y., Hamoudi R. (2020). Salivary microRNA 155, 146a/b and 203: A pilot study for potentially non-invasive diagnostic biomarkers of periodontitis and diabetes mellitus. PLoS ONE.

[38] Nisha K.J., Janam P., Harshakumar K. (2019). Identification of a novel salivary biomarker miR-143-3p for periodontal diagnosis: A proof of concept study. J. Periodontol..

[39] Ou L., Sun T., Cheng Y., Huang L., Zhan X., Zhang P., Yang J., Zhang Y., Zhou Z. (2019). MicroRNA-214 contributes to regulation of necroptosis via targeting ATF4 in diabetes-associated periodontitis. J. Cell Biochem..

[40] Amaral S.A., Pereira T.S.F., Brito J.A.R., Cortelli S.C., Cortelli J.R., Gomez R.S., Costa F.O., Miranda Cota L.O. (2019). Comparison of miRNA expression profiles in individuals with chronic or aggressive periodontitis. Oral. Dis..

[41] Zhang Y., Li S., Yuan S., Zhang H., Liu J. (2019). MicroRNA-23a inhibits osteogenesis of periodontal mesenchymal stem cells by targeting bone morphogenetic protein signaling. Arch. Oral. Biol..

[42] Zhao S., Cheng Y., Kim J.G. (2019). microRNA-146a downregulates IL-17 and IL-35 and inhibits proliferation of human periodontal ligament stem cells. J. Cell Biochem..

[43] Fujimori K., Yoneda T., Tomofuji T., Ekuni D., Azuma T., Maruyama T., Mizuno H., Sugiura Y., Morita M. (2019). Detection of Salivary miRNAs Reflecting Chronic Periodontitis: A Pilot Study. Molecules.

[44] Bagavad Gita J., George A.V., Pavithra N., Chandrasekaran S.C., Latchumanadhas K., Gnanamani A. (2019). Dysregulation of miR-146a by periodontal pathogens: A risk for acute coronary syndrome. J. Periodontol..

[45] Naqvi A.R., Brambila M.F., Martínez G., Chapa G., Nares S. (2019). Dysregulation of human miRNAs and increased prevalence of HHV miRNAs in obese periodontitis subjects. J. Clin. Periodontol..

[46] Yoneda T., Tomofuji T., Ekuni D., Azuma T., Maruyama T., Fujimori K., Sugiura Y., Morita M. (2019). Serum microRNAs and chronic periodontitis: A case-control study. Arch. Oral. Biol..

[47] Micó-Martínez P., García-Giménez J.L., Seco-Cervera M., López-Roldán A., Almiñana-Pastor P.J., Alpiste-Illueca F., Pallardó F.V. (2018). miR-1226 detection in GCF as potential biomarker of chronic periodontitis: A pilot study. Med. Oral. Patol. Oral. Cir. Bucal..

[48] Venugopal P., Koshy T., Lavu V., Ranga Rao S., Ramasamy S., Hariharan S., Venkatesan V. (2018). Differential expression of microRNAs let-7a, miR-125b, miR-100, and miR-21 and interaction with NF-kB pathway genes in periodontitis pathogenesis. J. Cell Physiol..

[49] Li J., Wang R., Ge Y., Chen D., Wu B., Fang F. (2019). Assessment of microRNA-144-5p and its putative targets in inflamed gingiva from chronic periodontitis patients. J. Periodontal Res..

[50] Ghotloo S., Motedayyen H., Amani D., Saffari M., Sattari M. (2019). Assessment of microRNA-146a in generalized aggressive periodontitis and its association with disease severity. J. Periodontal Res..

[51] Radović N., NikolićJakoba N., Petrović N., Milosavljević A., Brković B., Roganović J. (2018). MicroRNA-146a and microRNA-155 as novel crevicular fluid biomarkers for periodontitis in non-diabetic and type 2 diabetic patients. J. Clin. Periodontol..

[52] Saito A., Horie M., Ejiri K., Aoki A., Katagiri S., Maekawa S., Suzuki S., Kong S., Yamauchi T., Yamaguchi Y. (2017). MicroRNA profiling in gingival crevicular fluid of periodontitis-a pilot study. FEBS Open Bio.

[53] Na H.S., Park M.H., Song Y.R., Kim S., Kim H.J., Lee J.Y., Choi J.I., Chung J. (2016). Elevated MicroRNA-128 in Periodontitis Mitigates Tumor Necrosis Factor-α Response via p38 Signaling Pathway in Macrophages. J. Periodontol..

[54] Motedayyen H., Ghotloo S., Saffari M., Sattari M., Amid R. (2015). Evaluation of MicroRNA-146a and Its Targets in Gingival Tissues of Patients with Chronic Periodontitis. J. Periodontol..

[55] Kalea A.Z., Hoteit R., Suvan J., Lovering R.C., Palmen J., Cooper J.A., Khodiyar V.K., Harrington Z., Humphries S.E., D’Aiuto F. (2015). Upregulation of gingival tissue miR-200b in obese periodontitis subjects. J. Dent. Res..

[56] Ogata Y., Matsui S., Kato A., Zhou L., Nakayama Y., Takai H. (2014). MicroRNA expression in inflamed and noninflamed gingival tissues from Japanese patients. J. Oral. Sci..

[57] Kadkhodazadeh M., Jafari A.R., Amid R., Ebadian A.R., Alipour M.M., Mollaverdi F., Lafzi A. (2013). MiR146a and MiR499 gene polymorphisms in Iranian periodontitis and peri-implantitis patients. J. Long. Term. Eff. Med. Implant..

[58] Perri R., Nares S., Zhang S., Barros S.P., Offenbacher S. (2012). MicroRNA modulation in obesity and periodontitis. J. Dent. Res..

[59] Lee Y.H., Na H.S., Jeong S.Y., Jeong S.H., Park H.R., Chung J. (2011). Comparison of inflammatory microRNA expression in healthy and periodontitis tissues. Biocell.

[60] YXie F., Shu R., Jiang S.Y., Liu D.L., Zhang X.L. (2011). Comparison of microRNA profiles of human periodontal diseased and healthy gingival tissues. Int. J. Oral. Sci..

[61] M’Baya-Moutoula E., Louvet L., Meuth V.M.-L., Massy Z.A., Metzinger L. (2015). High inorganic phosphate concentration inhibits osteoclastogenesis by modulating miR-223. Biochim. Biophys. Acta Mol. Basis Dis..

[62] Zhang L., Lv H., Cui Y., Shi R. (2022). The role of long non-coding RNA (lncRNA) nuclear paraspeckle assembly transcript 1 (NEAT1) in chronic periodontitis progression. Bioengineered.

[63] Yang Q., Liu P., Han Y., Wang C., Huang Y., Li X., Zheng Y., Li W. (2022). Long noncoding RNA GAS5 alleviates the inflammatory response of human periodontal ligament stem cells by regulating the NF-κBsignalling pathway. Eur. J. Orthod..

[64] Han Y., Huang Y., Yang Q., Jia L., Zheng Y., Li W. (2022). Long non-coding RNA SNHG5 mediates periodontal inflammation through the NF-κBsignalling pathway. J. Clin. Periodontol..

[65] Wang X., Wang Y. (2021). LncRNA DCST1-AS1 inhibits PDLCs’ proliferation in periodontitis and may bind with miR-21 precursor to upregulate PLAP-1. J. Periodontal Res..

[66] Zhou M., Hu H., Han Y., Li J., Zhang Y., Tang S., Yuan Y., Zhang X. (2021). Long non-coding RNA 01126 promotes periodontitis pathogenesis of human periodontal ligament cells via miR-518a-5p/HIF-1α/MAPK pathway. Cell Prolif..

[67] Sayad A., Ghafouri-Fard S., Shams B., Arsang-Jang S., Gholami L., Taheri M. (2020). Sex-specific up-regulation of p50-associated COX-2 extragenic RNA (PACER) lncRNA in periodontitis. Heliyon.

[68] Li J., Wang M., Song L., Wang X., Lai W., Jiang S. (2020). LncRNA MALAT1 regulates inflammatory cytokine production in lipopolysaccharide-stimulated human gingival fibroblasts through sponging miR-20a and activating TLR4 pathway. J. Periodontal Res..

[69] Guo R., Huang Y., Liu H., Zheng Y., Jia L., Li W. (2020). Long Non-Coding RNA H19 Participates in Periodontal Inflammation via Activation of Autophagy. J. Inflamm. Res..

[70] Gholami L., Ghafouri-Fard S., Mirzajani S., Arsang-Jang S., Taheri M., Dehbani Z., Dehghani S., Houshmand B., Amid R., Sayad A. (2020). The lncRNA ANRIL is down-regulated in peripheral blood of patients with periodontitis. Noncoding RNA Res..

[71] Dong Y., Feng S., Dong F. (2020). Maternally-Expressed Gene 3 (MEG3)/miR-143-3p Regulates Injury to Periodontal Ligament Cells by Mediating the AKT/Inhibitory κB Kinase (IKK) Pathway. Med. Sci. Monit..

[72] Wang X., Ma F., Jia P. (2019). LncRNA AWPPH overexpression predicts the recurrence of periodontitis. Biosci. Rep..

[73] Liu Y., Liu C., Zhang A., Yin S., Wang T., Wang Y., Wang M., Liu Y., Ying Q., Sun J. (2019). Down-regulation of long non-coding RNA MEG3 suppresses osteogenic differentiation of periodontal ligament stem cells (PDLSCs) through miR-27a-3p/IGF1 axis in periodontitis. Aging.

[74] Liu W., Zheng Y., Chen B., Ke T., Shi Z. (2019). LncRNA papillary thyroid carcinoma susceptibility candidate 3 (PTCSC3) regulates the proliferation of human periodontal ligament stem cells and toll-like receptor 4 (TLR4) expression to improve periodontitis. BMC Oral. Health.

[75] Chen P., Huang Y., Wang Y., Li S., Chu H., Rong M. (2019). MALAT1 overexpression promotes the proliferation of human periodontal ligament stem cells by upregulating fibroblast growth factor 2. Exp. Ther. Med..

[76] Chen H., Lan Z., Li Q., Li Y. (2019). Abnormal expression of long noncoding RNA FGD5-AS1 affects the development of periodontitis through regulating miR-142-3p/SOCS6/NF-κB pathway. Artif Cells Nanomed. Biotechnol..

[77] Wang L., Wu F., Song Y., Li X., Wu Q., Duan Y., Jin Z. (2016). Long noncoding RNA related to periodontitis interacts with miR-182 to upregulate osteogenic differentiation in periodontal mesenchymal stem cells of periodontitis patients. Cell Death Dis..

[78] Wang J., Wang Z., Huang M., Zhang Y., Xu L. (2023). Circ_0099630 Participates in SPRY1-Mediated Repression in Periodontitis. Int. Dent. J..

[79] Pan J., Zhao L., Liu J., Wang G. (2022). Inhibition of circular RNA circ_0138959 alleviates pyroptosis of human gingival fibroblasts via the microRNA-527/caspase-5 axis. Bioengineered.

[80] Wei Y., Peng Z. (2022). Hsa_circ_0099630 knockdown induces the proliferation and osteogenic differentiation and attenuates the apoptosis of porphyromonasgingivalis lipopolysaccharide-induced human periodontal ligament fibroblasts. Ann. Transl. Med..

[81] Zhao X.Q., Ao C.B., Yan Y.T. (2022). The circular RNA circ_0099630/miR-940/receptor-associated factor 6 regulation cascade modulates the pathogenesis of periodontitis. J. Dent. Sci..

[82] Wang J., Du C., Xu L. (2021). Circ_0081572 inhibits the progression of periodontitis through regulating the miR-378h/RORA axis. Arch. Oral. Biol..

[83] Yu B., Hu J., Li Q., Wang F. (2021). CircMAP3K11 Contributes to Proliferation, Apoptosis and Migration of Human Periodontal Ligament Stem Cells in Inflammatory Microenvironment by Regulating TLR4 via miR-511 Sponging. Front. Pharmacol..

[84] Zheng J., Zhu X., He Y., Hou S., Liu T., Zhi K., Hou T., Gao L. (2021). CircCDK8 regulates osteogenic differentiation and apoptosis of PDLSCs by inducing ER stress/autophagy during hypoxia. Ann. N. Y. Acad. Sci..

[85] Li J., Xie R. (2019). Circular RNA expression profile in gingival tissues identifies circ_0062491 and circ_0095812 as potential treatment targets. J. Cell Biochem..

[86] Wang F., Chen X., Han Y., Xi S., Wu G. (2019). circRNA CDR1as Regulated the Proliferation of Human Periodontal Ligament Stem Cells under a Lipopolysaccharide-Induced Inflammatory Condition. Mediat. Inflamm..

[87] Luan X., Zhou X., Naqvi A., Francis M., Foyle D., Nares S., Diekwisch T.G.H. (2018). MicroRNAs and immunity in periodontal health and disease. Int. J. Oral. Sci..

[88] Santonocito S., Polizzi A., Palazzo G., Isola G. (2021). The Emerging Role of microRNA in Periodontitis: Pathophysiology, Clinical Potential and Future Molecular Perspectives. Int. J. Mol. Sci..

[89] Ru L., Pan B., Zheng J. (2023). Signalling pathways in the osteogenic differentiation of periodontal ligament stem cells. Open Life Sci..

[90] Micó-Martínez P., Almiñana-Pastor P.J., Alpiste-Illueca F., López-Roldán A. (2021). MicroRNAs and periodontal disease: A qualitative systematic review of human studies. J. Periodontal Implant. Sci..

[91] Xu J., Yin Y., Lin Y., Tian M., Liu T., Li X., Chen S. (2021). Long non-coding RNAs: Emerging roles in periodontitis. J. Periodontal Res..

[92] Kristensen L.S., Andersen M.S., Stagsted L.V.W., Ebbesen K.K., Hansen T.B., Kjems J. (2019). The biogenesis, biology and characterization of circular RNAs. Nat. Rev. Genet..

[93] Jiao K., Walsh L.J., Ivanovski S., Han P. (2021). The Emerging Regulatory Role of Circular RNAs in Periodontal Tissues and Cells. Int. J. Mol. Sci..

[94] Ghafouri-Fard S., Dashti S., Gholami L., Badrlou E., Sadeghpour S., Hussen B.M., Hidayat H.J., Nazer N., Shadnoush M., Sayad A. (2022). Expression analysis of Wnt signaling pathway related lncRNAs in periodontitis: A pilot case-control study. Hum. Gene..

[95] Sayad A., Mirzajani S., Gholami L., Razzaghi P., Ghafouri-Fard S., Taheri M. (2020). Emerging role of long non-coding RNAs in the pathogenesis of periodontitis. Biomed. Pharmacother..

[96] Wang S., Duan Y. (2022). LncRNA OIP5-AS1 inhibits the lipopolysaccharide-induced inflammatory response and promotes osteogenic differentiation of human periodontal ligament cells by sponging miR-92a-3p. Bioengineered.

[97] Sufianov A., Beilerli A., Begliarzade S., Ilyasova T., Kudriashov V., Liang Y., Beylerli O. (2023). The role of noncoding RNAs in the osteogenic differentiation of human periodontal ligament-derived cells. Non-Coding RNA Res..

[98] Xi X.U., Lang G.P., Chen Z.L., Le Wang J., Han Y.Y. (2023). The Dual Role of Non-coding RNAs in the Development of Periodontitis. Biomed. Environ. Sci..

[99] AlRowis R., AlMoharib H.S., AlMubarak A., Bhaskardoss J., Preethanath R.S., Anil S. (2014). Oral fluid-based biomarkers in periodontal disease—Part 2. Gingival crevicular fluid. J. Int. Oral. Health.

[100] Baliban R.C., Sakellari D., Li Z., Guzman Y.A., Garcia B.A., Floudas C.A. (2013). Discovery of biomarker combinations that predict periodontal health or disease with high accuracy from GCF samples based on high-throughput proteomic analysis and mixed-integer linear optimization. J. Clin. Periodontol..

[101] Asa’ad F., Garaicoa-Pazmiño C., Dahlin C., Larsson L. (2020). Expression of MicroRNAs in Periodontal and Peri-Implant Diseases: A Systematic Review and Meta-Analysis. Int. J. Mol. Sci..

[102] Belgardt B.F., Ahmed K., Spranger M., Latreille M., Denzler R., Kondratiuk N., von Meyenn F., Villena F.N., Herrmanns K., Bosco D. (2015). The microRNA-200 family regulates pancreatic beta cell survival in type 2 diabetes. Nat. Med..

[103] (1999). 1999 International International Workshop for a Classification of Periodontal Diseases and Conditions. Papers. Oak Brook, Illinois, 30 October–2 November 1999. Ann. Periodontol..

[104] Papapanou P.N., Sanz M., Buduneli N., Dietrich T., Feres M., Fine D.H., Flemmig T.F., Garcia R., Giannobile W.V., Graziani F. (2018). Periodontitis: Consensus report of workgroup 2 of the 2017 World Workshop on the Classification of Periodontal and Peri-Implant Diseases and Conditions. J. Periodontol..

